# Diseases of the Rectum and Anus

**Published:** 1896-12

**Authors:** George K. Sims

**Affiliations:** Richmond, Va.; Adjunct Professor of Clinical Surgery, University College of Medicine; 621 Franklin Street


### Southern Medical Record.

A MONTHLY JOURNAL OF MEDICINE AND SURGERY.

VOL. XXVI. ATLANTA, GA., DECEMBER, 1896. No. 12.

### Original Articles.

### DISEASES OF THE RECTUM AND ANUS.*

BY GEORGE K. SIMS, M. D., RICHMOND, VA.,

Adjunct Professor of Clinical Surgery, University College of Medicine.

Formerly this class of diseases received but little attention by the profession; consequently quacks fell heir to a great many of them. In recent years they have received more attention, but the majority of the profession do not give them as much attention as they merit. There is no class of diseases that cause more annoyance and suffering, or that patients will be more grateful for curing them, or more willing to pay for. Time will permit me to give only a general outline of the most important of them to-night.

A *careful physical examination* should be made in every case. The dorsal is the best position for external inspection and eversion of the anal margin; for parts higher up and for operation, the knee-chest and the lateral or Sims' position is better. A female should always be placed in the Sims position, for obvious reasons.

*Read before Richmond Academy of Medicine and Surgery, October 13, 1896.

In making the examination, the tissues surrounding the anus should first be carefully inspected; after this, the interior of the rectum must be examined. This can be done either with the finger or the speculum, or both. A digital examination will be more agreeable to the patient if the finger is well lubricated and introduced gently. The best lubricant for the finger that I have used is ordinary toilet soap; it fills the cracks around and under the nail and prevents the fecal matter from sticking. This can be easily washed out, and you get rid of the bad odor, which is a very important consideration. If there is much soreness about the parts, carbolized vaseline will be much more agreeable to the patient.

In introducing the finger, notice the amount of resistance offered by the sphincters. The internal is an involuntary and the external is a voluntary muscle; they are separated by a well-marked interspace. A firmly contracted sphincter is met with in cases of fissure ani; a loosely contracted one may be due to atony or paralysis, induced by repeated stretchings by a polypus, hemorrhoids, or prolapsus. After passing the sphincter, the finger enters a large cavity called the rectal pouch, which is apparently without shape or form; if empty, the anterior and posterior walls are in contact. A great aid to digital examination is to fill the rectum with air or water, introduced by an ordinary bulb syringe. This puts the wall of the rectum on the stretch, so that any diseased condition can more easily be detected. After the digital examination, it may be necessary, for purposes of diagnosis or treatment, to introduce the speculum; in many cases it will be necessary to give an anesthetic to do this, or even to insert the finger.

1. *Hemorrhoids.*—The first subject I wish to review is *hemorrhoids.* Of these there are two varieties—external and internal—depending on their relation to the sphincter. *External piles* are venous or cutaneous. Of the former there are two kinds; first, a varicose condition of the external hemorrhoidal veins; this is very common and usually requires no treatment. The second is the thrombic pile, due to a thrombus in the external hemorrhoidal veins. A *thrombic pile* is tense, hard, and very painful; it usually comes on suddenly, and the bluish appearance of the thrombus can often be seen. The treatment may be palliative, which consists in keeping the stools very soft by laxatives and enemas, and the local application of soothing lotions or unguents, such as lead and opium wash, fluid hydrastis or hammamelis and lead water, or unguents of belladonna, opium, etc., followed by warm fomentations. If the size of the tumor or the pain seem to justify it, immediate relief may be given by incising the pile and turning out the clot—then applying a pad of antiseptic gauze and firm pressure by a "T" bandage to prevent the cavity from refilling.

Of *cutaneous piles*, authors recognize three varieties: First, redundant cutaneous piles. These are common in persons having internal piles, and are said to be diagnostic of that condition, with weakening of the internal sphincter. The second form is the hyperplastic pile; this is due to a hyperplasia of the connective tissue, from abrasions, fissure or ulceration. It is most often seen at the posterior border of the anus, in connection with fissure ani—in fact, it is pathognomonic of that disease. The third form is a hypertrophy of the normal radiating folds of the anus, the result of eczematous inflammation. The treatment of these conditions consists in removing the cause; then, if necessary, a portion of the redundant tissue may be excised and treated antiseptically. External piles should never be injected, as it can do no good and causes unnecessary torture.

*Internal hemorrhoids* are vascular tumors—composed of dilated veins, capillaries and small arterioles. Ordinarily, they lie just within the anal opening, but they may extend as high as two inches from the anus; they may be single or multiple. They are caused by an increase of blood pressure about the rectum, with obstruction to the return circulation, arising from diseases of the heart, lungs or liver, enlarged prostrate, stricture of the urethra, stone in the bladder, phymosis, cancer, or stricture of the rectum, and chronic constipation. Symptoms: a feeling of weight, itching, tenesmus, and pain, which may radiate to the genital or other pelvic organs. At first these are slight, but they gradually grow worse. The piles begin to protrude when at stool; later, a portion of the mucous membrane may prolapse. If they are not replaced, the contraction of the sphincter muscle will cause them to become swollen and congested, and gangrene may result. Rupture of the thin mucous membrane covering them causes hemorrhage—this may be very slight, or be several ounces. The blood is much redder than blood which escapes higher up than the rectum. The bleeding often relieves the pain and local symptoms for a time; but if it occurs frequently, as it often does, may cause serious anæmia. In long standing cases, the sphincter becomes so relaxed that it allows the protrusion of the piles to exist almost constantly.

The diagnosis, when they are protruding, is simple enough; but when they are not filled with blood your finger may scarcely feel them. In that case, after emptying the rectum with an enema, make the patient bear down as if at stool; at the same time draw the anus open with your fingers. They may now be seen or felt as they become distended. The smooth folds of mucous membrane of prolapse will hardly be mistaken for piles, as they lack the bunched appearance and the purplish color.

Rectal polypus, when protruding, is harder than piles or prolapse, and has a distinct pedicle.

*Treatment.*—The majority of cases can be made very comfortable, if taken in time, by palliative measures; they consist in keeping the bowels open by diet, exercise, salines, laxatives and enemas. Try to remove the cause by appropriate treatment. Locally use astringent injections and unguents; these should be pushed well up into the rectum. When these measures fail to give relief, a more radical treatment becomes necessary. Moderately severe cases may at times be cured by dilatation of the sphincter by means of the two thumbs. This requires an anesthetic, and must be done thoroughly. When the patient cannot take chloroform, or remain in bed for a few days, a cure may be effected by injecting a few drops of carbolic acid into the centre of each pile, treating only one at a time. This is tedious and uncertain at best, and not to be recommended. A better method of treating such cases is the one described by Dr. Earle, of Baltimore; this consists in injecting cocaine, applying a clamp, cutting off the pile, and stitching the edges together with a continuous suture of catgut. By treating only one pile at a time by this method, the patient need not lose any time from business.

When, however, the piles are large and numerous, they had better be all removed at one sitting, by one of the standard operations, which are the clamp and cautery, ligation, and Whitehead's. Of the three, the clamp and cautery is the one most generally used, being at the same time simple, safe, and effective. I will not enter into the details of these operations, but will mention a few practical points in the management. The patient should have a brisk cathartic two days before, and an enema the day before, and on the day of operation. The sphincter should be well stretched and temporarily paralyzed at the time of operation. No packing is necessary afterwards except a good external pad and a "T" bandage.

The bowels can be removed on the third or fourth day with salines or enemata. The patient must be kept in bed five or six days and in a week can be discharged.

2. *Anal fistulas* may be complete or incomplete—the latter are external and internal. They result from a peri-rectal or an ischio-rectal abscess, which was not opened early enough to prevent the formation of an orifice in the bowel. The sinus is prevented from healing by the passage of fecal matter and gases, which keep up the irritation. The fistulous track is usually tortuous, and there are frequently blind sinuses leading off from it into the cellular tissues surrounding the rectum. Sometimes it goes around the bowel and comes out on the opposite side, forming a horseshoe fistula. An internal sinus, if left to nature, will sooner or later make an opening on the skin; but it may burrow into the ischio-rectal fossa a long time before it does so.

The *diagnosis* is easily made when the external or internal opening is found, but it is often difficult to find the internal opening. It will usually be found within the grasp of the internal sphincter—occasionally higher up. The sinus may extend above the internal opening, and rarely a second opening is present. If the probe, carefully passed into the sinus, fails to find the internal opening, inject some hydrogen dioxide into it; it will frequently find its way through and can be seen foaming in the rectum. It must be remembered that a sinus opening far down the thigh may lead to the rectum. On the other hand, we should not forget that a sinus in the neighborhood of the anus may be due to caries of the tuberosity of the ischium, or coccyx, or even hip-joint disease, and have no connection with the rectum. In incomplete fistules there is an intermittent discharge of pus from the anus, especially when pressure is made upon the circum-anal integument.

*Treatment.*—Injecting the fistule with stimulating solutions is usually unavailing. An operation is almost always necessary to make a cure. Fistulæ resulting from stricture or malignant disease of rectum, should not be operated upon unless the primary cause can be removed. All abscesses in this region should be opened early, curetted and treated antiseptically; this can often be done at the office, under cocaine. A very good method of treating many cases is the elastic ligature; draw it tight and allow the patient to go about his business. The pain is quite severe for the first day or two. In three to seven days the ligature will cut through, and while it is cutting the tract is beginning to granulate behind it so that, when it has cut through, two-thirds of the tract will be healed. The rest of it will heal after a few applications of silver nitrate, and aristol, iodoform or loretin. The ideal treatment is to incise all the structures between the two openings on a grooved director, also lay open any communicating sinuses; the fistulous tract should then be scraped out with a curette, or dissected out, washed out with a 1.3000 bichloride solution, and the walls brought together by catgut sutures. Aristol or loretin should then be applied, and the bowel confined for four or five days. In incomplete internal fistulæ, find the orifice, then pass a grooved director from within, against the skin, make an opening here, then incise all the structures, and proceed as in the complete fistulæ. When it is impossible to clean out the fistulous tract thoroughly, they should be washed out with hydrogen dioxide then kept packed with iodoform gauze to make them heal up from the bottom. The rectum should be washed out with an antiseptic solution after every evacuation and fresh gauze packing applied.

3. *Anal fissure* is a linear ulcer situated just within the verge of the anus. It results from a tear or excoriation in the mucous membrane of the sphincter, caused by hardened feces, syphilis, or the irritating discharges of diarrhœa or dysentery. It fails to heal because of the frequent irritation by fecal matter, and the motion and spasm of the sphincter.

The *symptoms* of anal fissure are remarkable for the severity of the pain, which is intense, notwithstanding the insignificance of the lesion. It comes on during defecation, and may last for many hours. The manner in which it is reflected may cause the patient to attribute his suffering to disease of the bladder, urethra or other pelvic organs. The dread of the intense pain causes the patient to refrain from emptying the rectum, and constipation results. Spasm of the sphincter muscle is the cause of this agonizing pain, and is always present in true anal fissure. It is often so great that it is impossible to pass the finger into the rectum, or to make a satisfactory examination without an anesthetic.

*Treatment.*—In recent cases, a cure can often be effected by keeping the feces soft by laxatives and enemata, washing out the rectum with carbolic acid or other antiseptic solution after each evacuation, the application of silver nitrate and some antiseptic powder of unguent to the ulcer. When this treatment fails, as it often will in chronic cases, the next best treatment is to thoroughly paralyze the sphincter by stretching it with the thumbs. Complete anesthesia is required for this operation. Another method, said to be more effectual than dilation, is to make an incision through the base of the ulcer, dividing either the whole or a portion of the sphincter. Any small tab of mucous membrane of polypoid growth complicating the fissure, should be removed at the time of operation.

4. *Prolapse of the rectum* may be partial when the mucous membrane only protrudes, or complete when all its coats are involved, the rectum being turned inside out. The disease is most common in children and the aged, from a weakened condition of the tissues. Anything that causes abnormal straining and bearing down—as stone in the bladder, urethral stricture, polypus, dysentery, chronic constipation, or phymosis—may be the exciting cause.

The *diagnosis* is usually very simple; the prolapsed tissues appear at first only when at stool, but frequent repetition of the process soon weakens the sphincter so that the prolapse occurs whenever the patient walks or assumes the erect posture. If allowed to remain out long, the tissues become so much swollen that it is difficult to reduce, and strangulation and gangrene may result.

*Treatment.*—The prolapsed tissues must be reduced by gentle though firm pressure with the fingers, using some lubricant on the parts. During the reduction, the patient should assume the knee-chest or the lateral (Sims') position. If these measures fail, cover the finger with a piece of lint and insert it into the orifice of the protruded bowel and push it in slowly and gently. After it is reduced, the finger is withdrawn, and subsequently the lint pulled out. The rectum must be supported by a "T" bandage or strips of adhesive plaster, applied so as to hold the nates close together to prevent recurrence. The bowels must be kept soft, and any trouble that is likely to produce straining must be removed. The patient should lie on the side or back or stand while evacuating the bowels. Astringent enemas, ointments and suppositories are serviceable.

Under such a line of treatment, cases occurring in children and cases of moderate severity in adults, can be cured. The more serious cases require surgical treatment. With a thermo-cautery make several longitudinal applications to the prolapsed tissues. Another method is to clamp longitudinal portions of the mucous membrane, which is then cut off and the stump seared with a red-hot cautery iron. In chronic cases, in which the sphincter has become relaxed, the triangular portion of the sphincter, and also a portion of the posterior wall of the rectum may be removed; sutures are then applied so as to bring the divided walls together.

5. *Ulceration of the rectum* may be dysenteric, syphilitic, tuberculous, or malignant. The exciting cause is often constipation. It is most common among the aged, and is often mistaken for dysentery. Symptoms: irregular diarrhœa, pain, tenesmus, muco-purulent, and blood-stained discharges and other symptoms of dysentery. Ulceration in the upper part of the rectum is far less painful than in the lower part, within the grasp of the sphincter.

*Treatment.*—Keep the excreta soft, wash out the rectum after each stool, and use mild, astringent, antiseptic and anodyne enemas and suppositories. A weak carbolic solution is very soothing and healing. An enema of laudanum in starch water will relieve the pain. Tubercular ulcers require iodoform, and syphilitic cases require the iodides and mercury, in addition to the local treatment.

*621 Franklin Street.*

